# A single scalp nodule as the first presentation of acute lymphoblastic leukemia (*KMT2A::MLLT3*) in a healthy-appearing infant: a case report

**DOI:** 10.3389/fped.2023.1254274

**Published:** 2023-12-08

**Authors:** Francesco Pellegrino, Paola Coppo, Elena Barisone, Nicoletta Bertorello, Manuela Spadea, Franca Fagioli

**Affiliations:** ^1^ Department of Pediatric and Public Health Sciences, Postgraduate School of Pediatrics, Regina Margherita Children Hospital, University of Turin, Turin, Italy; ^2^ Pediatric Dermatology, Regina Margherita Children’s Hospital, Città della Salute e della Scienza di Torino, Torino, Italy; ^3^ Stem Cell Transplantation and Cellular Therapy Laboratory, Paediatric Onco-Haematology Division, Regina Margherita Children’s Hospital, City of Health and Science of Turin, Torino, Italy

**Keywords:** leukemia cutis, acute lymphoblastic leukemia, scalp nodule, ALL, leukemia

## Abstract

**Background:**

Infant leukemia is a rare form of acute leukemia diagnosed prior to the age of 1 and is characterized by an extremely poor prognosis due to its dismal response to current therapeutic approaches. It comprises about 4% of all childhood cases of acute lymphoblastic leukemia (ALL). Isolated initial cutaneous involvement in ALL is uncommon, and even more so in infant ALL.

**Case presentation:**

Here, we present the case of a 2-month-old healthy-appearing infant, initially presenting with a single scalp nodule and subsequently diagnosed with an infant ALL. The leukemia was characterized by the most immature B-lineage immunophenotype [pro-B ALL/B-I, according to the European Group for the Immunological Characterization of Leukaemias (EGIL) classification] and chromosomal translocation t(9;11)(p22;q23), resulting in fusion gene *KMTLA2::MLLT3*, which is considered a negative prognostic factor. The patient underwent hematopoietic stem cell transplantation and is still in remission.

**Conclusions:**

This case is peculiar because of the rare occurrence of isolated initial cutaneous involvement in ALL. Despite the healthy appearance of the patient, every suspicious symptom suggestive of malignancies should be further investigated to anticipate the diagnosis and start treatment early.

## Introduction

Infant leukemia refers to acute leukemia diagnosed prior to the age of 1 and is a rare aggressive type of leukemia. It comprises about 4% of all childhood cases of acute lymphoblastic leukemia (ALL). There is a slight predominance of lymphoid cases over myeloid cases in infant leukemia, and of the lymphoid cases, nearly all belong to the B-cell lineage, with <5% classified as T-cell lineage (1). Infant ALL is more often associated with a very immature B-cell immunophenotype without CD10 expression [pro-B ALL/BI, according to the European Group for the Immunological Characterization of Leukaemias (EGIL) classification] (2, 3). Compared with older children, infants with acute leukemia tend to present with more aggressive features, including elevated white blood cell (WBC) count, hepatosplenomegaly, central nervous system (CNS) involvement, and skin infiltration, commonly referred to as leukemia cutis (LC) (4). We present the case of a 2-month-old healthy-appearing infant, who was referred to our dermatological department for evaluation of a scalp nodule; subsequent investigations established the diagnosis of infant ALL. It was characterized by the most immature B-lineage immunophenotype (pro-B ALL/B-I, according to EGIL classification) and chromosomal translocation t(9;11)(p22;q23), resulting in fusion gene *KMTLA2::MLLT3*. This case is peculiar because of the uncommon occurrence of isolated initial cutaneous involvement in ALL and even more so in infant ALL. Moreover, chromosomal translocation t(9;11)(p22;q23) is mainly associated with acute myeloid leukemia (AML) and occurs only in a small percentage of ALL (5).

## Case presentation

**Figure 1:**
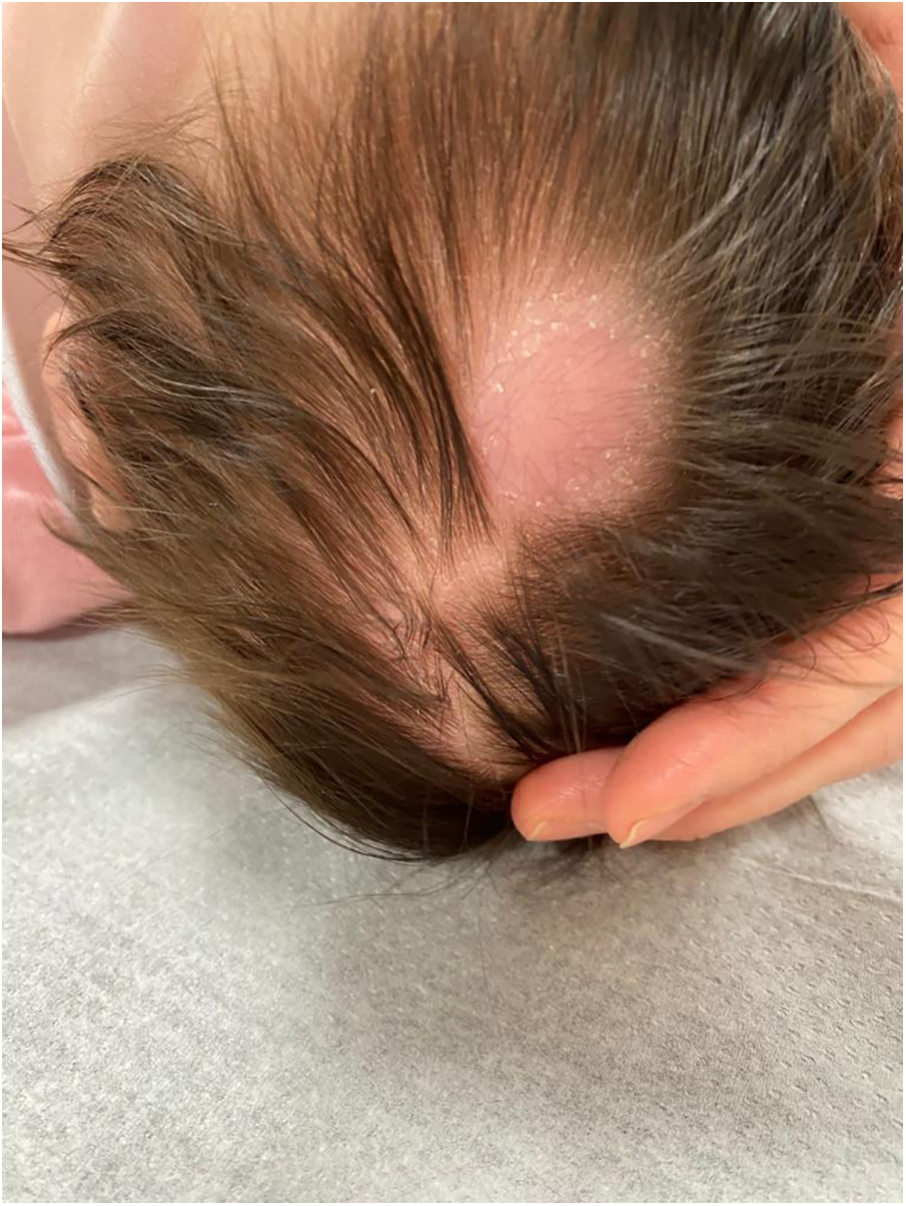
Patient at 2 months of age with a primarily isolated scalp nodule.

**Figure 2:**
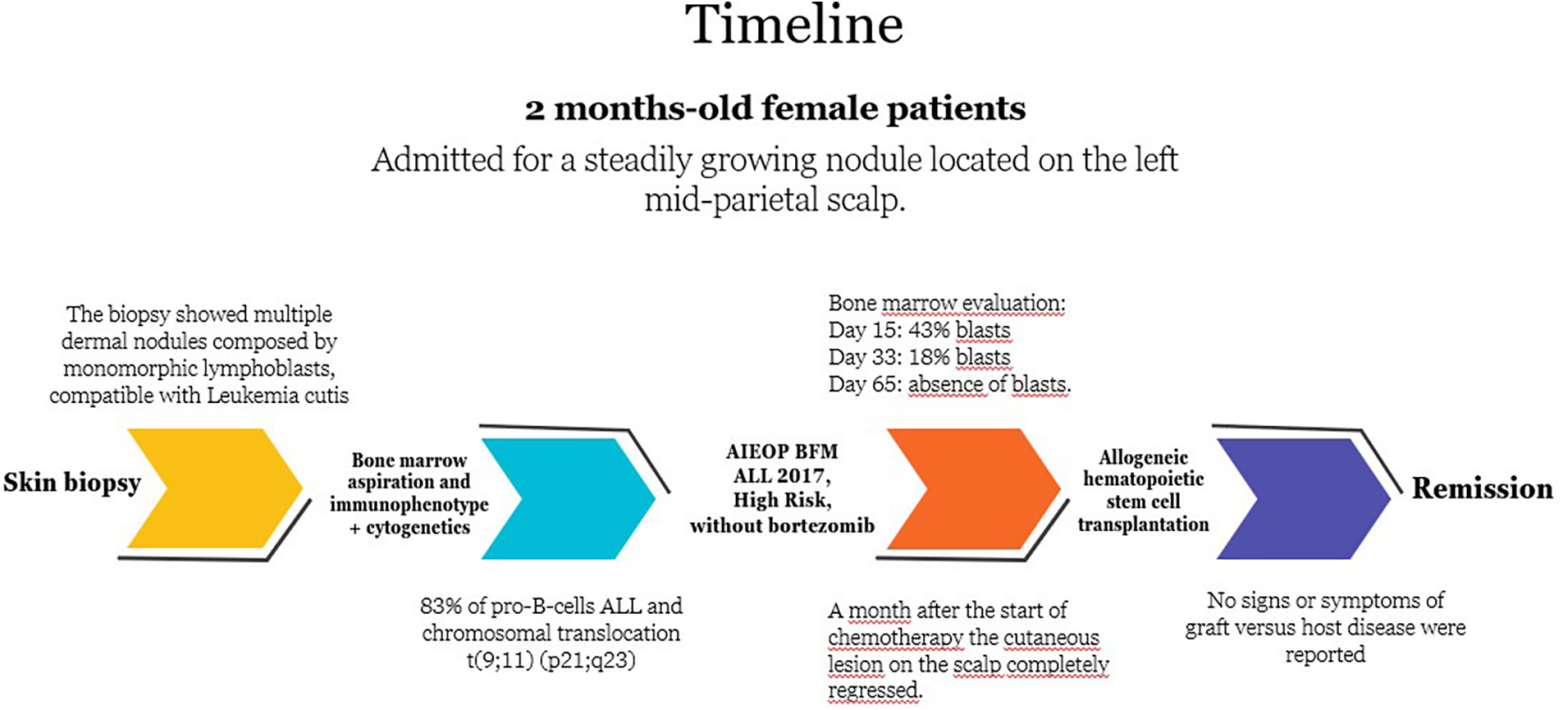
Timeline with relevant data from the episode of care.

A 2-month-old asymptomatic female infant presented to our dermatological department, complaining of a steadily growing nodule located on the left mid-parietal scalp (Figure 1). It appeared as a mobile, well-circumscribed, smooth swelling, forming an alopecic area measuring approximately 3 cm × 3 cm. No dysmorphic features or other remarkable clinical signs or symptoms were observed. Lymphadenopathy and hepatosplenomegaly were not observed. Her birth and family history were unremarkable, and her nutritional status was good. On suspecting malignancies, dermatologists performed a punch skin biopsy from the scalp nodule. The biopsy results revealed multiple dermal nodules composed of monomorphic lymphoblasts, diffusely infiltrating the dermis and subcutis, consistent with LC. Immunohistochemical staining revealed positive expression of CD22, CD19, CD34, and terminal deoxynucleotidyl transferase (TdT) in the nodules, which points toward a B-cell lineage. Meanwhile, laboratory examination showed a white blood cell count of 21,940/µl, a hemoglobin level of 7.6 g/dl, and a platelet count of 38,300/µl. The peripheral blood morphologic evaluation showed 45% circulating blasts. A bone marrow aspiration revealed 88% blast cells, with rare myeloid cells and lymphocytes upon morphological examination, while the bone marrow immunophenotype confirmed the presence of 83% of pro-B/B-I cells in ALL (with positive expression of CD19, cyCD22, and cyCD79a and negative expression of CD10), without any coexpression of myeloid markers together with B-lineage antigens. Cytogenetic analysis performed on bone marrow specimens detected the presence of chromosomal translocation t(9;11) (p21;q23). Cerebrospinal fluid analysis did not reveal leukemic infiltration of the central nervous system. The patient was subsequently treated according to protocol AIEOP BFM ALL 2017, high-risk (HR) pB-ALL (6). The immunophenotype of bone marrow on day 15 after the start of the treatment showed the presence of 43% blasts, which reduced to 18% on day 33. A month after the start of chemotherapy, the cutaneous lesion on the scalp completely regressed. The bone marrow evaluation by flow cytometry and molecular biology on day 65 confirmed the absence of blasts. The patient underwent Extended Consolidation (Consolext) for early HR pB-ALL in the R-eHR control arm, followed by Intensified Consolidation: Block HR-1′ followed by HR-2′ and HR-3′ (control arm in randomization R-HR). The patient was randomized without bortezomib and blinatumomab (6). Six months after the diagnosis, the patient finally received allogeneic hematopoietic stem cell transplantation (HSCT) with an HLA-identical matching donor after a preparatory induction regimen with treosulfan–fludarabine–thiotepa. No signs or symptoms of graft vs. host disease were reported. She developed hypertension, which is well controlled with antihypertensive drugs. She is now 20 months old and is still in remission 12 months post-HSCT. The timeline with relevant data from the episode of care is reported in Figure 2.

## Discussion

ALL is a neoplastic disease characterized by the abnormal proliferation of immature lymphoid cells. It accounts for 25%–30% of all cancer diagnoses among children (1).

“Infant leukemia” refers to acute leukemia diagnosed prior to the age of 1 and is a rare disease with an extremely poor prognosis due to its aggressive clinical presentation in a uniquely vulnerable host, poor response to current therapeutic approaches, and fascinating biology (4). LC describes the localized or disseminated infiltration of the epidermis and/or dermis by leukemic cells (7). The exact mechanism is not clear, but recent molecular analyses suggest the involvement of various chemokines, influencing cell–cell interaction and adhesion molecules to mediate the migration of leukemic cells via skin-selective homing processes (8).

We reported this unusual case because LC is usually seen in AML, while there are few reported cases of cutaneous involvement in children with ALL and even fewer in infant ALL (1, 9). The frequency of LC is 10%–15% in AML and only about 1% in ALL (8). Bone marrow infiltration and peripheral blood involvement generally develop prior to the appearance of cutaneous lesions; however, rarely, LC may be the primary manifestation of leukemia, preceding the systemic symptoms by months (7).

Bontoux et al. reported that in 11 children (31%) of their cohort of 38 patients affected by ALL with LC, skin lesions appeared before the ALL diagnosis, suggesting that cutaneous manifestations may help anticipate the diagnosis. That case series also confirmed that, although LC lesions may have a highly variable clinical presentation, the most common manifestations are erythematous or violaceous papules or nodules, involving principally the head or the trunk (10).

Other primary lesions described include macules, ulcers, bullae, and urticarial wheals, in addition to non-specific skin involvement resulting from bone marrow failure, such as petechiae, purpura, and ecchymoses.

Generally, the clinical manifestation of skin lesions is not correlated to a particular type of leukemia, except for gingival hypertrophy, mostly associated with AML and chloroma, which presents as a firm nodule with a greenish color, which is a peculiarity of AML (11).

An unusual scalp location similar to that presented by our patient has already been reported by Millot et al. in the largest series of children with ALL presenting with cutaneous involvement (12). Therefore, not only multiple nodules but also solitary scalp masses in pediatric patients should be considered suspicious for malignancy, and a skin biopsy should be performed to optimize differential diagnosis (13) (Table 1).

**Table 1:** Differential diagnosis of scalp nodules in infancy.

Nodule	Clinical characteristics
Congenital hemangioma	Vascular lesions that are fully formed at birth and remain stable. They are usually round or oval, elevated, and warm to the touch, with a parenchymatous consistency. They are dark pink to blue or purple
Deep infantile hemangioma	Vascular lesions that appear in the first weeks of life. They manifest as partially compressible, subcutaneous, bluish vascular swellings. They have a rapid proliferative phase in infancy, followed by a gradual involutional phase over the next several years of life. Doppler ultrasound study usually shows high vascular flow
Dermoid cyst	It is a growth of normal tissue enclosed in a pocket of cells, called a sac. It appears as a compressible, subcutaneous nodule with variable mobility, commonly on the superolateral orbit rim
Eosinophilic granuloma	It is the mildest form of Langerhans cell histiocytosis. The lesions are typically raised, clearly defined, and yellowish-pink. Pain and swelling in the region of involved bone is the most common presenting symptom
Juvenile xanthogranuloma	It is a non-Langerhans cell histiocytosis that is usually benign and self-limiting. The lesions are smooth, round, firm papules that change from red-brown to yellow
Leukemia cutis	It manifests as pink/red to tan nodules or plaques (with petechiae or purpura associated, according to bone marrow involvement)
Nevus psiloliparus	It is a rare fatty tissue nevus often described as a smoothly surfaced, irregularly shaped, circumscribed hairless lesion involving the scalp

Another peculiarity of our case is the detection of translocation t(9;11)(p22;q23) involving histone lysine methyltransferase 2A gene (*KMT2A*), formerly known as mixed-lineage leukemia (MLL), mainly associated with AML and extremely rare in ALL (14).

Over 90 *KMT2A* fusion partners have been identified until now, with MLLT3 and AFF1 as the most recurring ones, but the presence of *KMT2A* rearrangements is associated with a poor prognosis in ALL, independently from the partner gene (15).

A high number of infant leukemias are characterized by the *KMT2A::MLLT3* gene fusion. To better understand the pathogenetic role of *KMT2A* in malignant hematopoiesis, Hess et al. generated a *KMT2A* knockout mouse and found defective yolk sac hematopoiesis in *KMT2A*-null mice and a block in hematopoietic differentiation in *KMT2A*-null embryonic stem cells. These observations support the pivotal role of the *KMT2A::MLLT3* gene in the regulation of hematopoietic differentiation and leukemogenesis as well (16). Also, *KMT2A::MLLT3* gene rearrangements can be detected in blood samples at birth in most infant leukemia patients, suggesting a prenatal origin of leukemic cells in infant leukemia (17).

The different chromosomal rearrangements involving the *KMT2A::MLLT3* gene are associated with specific leukemia subtypes. For example, translocation t(4;11) (q21;q23), which generates the –*AFF1* fusion, is found predominantly in ALL (18), suggesting that the *AF4* segment has a role in controlling the lineage. On the other hand, translocation t(9;11) (p22;q23) is mainly associated with AML and fuses *MLLT3* to *KMT2A*, suggesting a role for *MLLT3* in myeloid disease (19).

Chen et al. showed a direct relationship between cell-specific transformation susceptibility and oncogene dosage effects in progenitor cells. The expression of the *KMT2A::MLLT3* fusion gene in common lymphoid progenitors (CLPs) rather than in granulocyte–monocyte progenitors (GMPs) results in a more aggressive disease associated with distinct origin-related gene-expression profiles (20). Thus, this translocation, without any other cytogenetic anomalies, is not a significant risk factor in infant AML, while it is clearly associated with poorer outcomes in infant ALL, such as elevated WBC counts at diagnosis and age less than 6 months (21). Although our patient presented several of these risk factors (age less than 6 months and chromosomal translocation), she remained in complete remission at 16 months from the start of chemotherapy.

## Conclusion

This case highlights the importance of an early diagnosis of ALL, demonstrating that cutaneous involvement in ALL could be the only physically detectable sign in a healthy-appearing infant, and any such manifestation should always be investigated. In fact, the early recognition of suspicious signs of leukemia may allow us to achieve precocious diagnosis and start therapies earlier, thus potentially improving prognoses in children, even in the presence of high-risk criteria.

## Data Availability

The original contributions presented in the study are included in the article/Supplementary Material, further inquiries can be directed to the corresponding author.
